# 

**Published:** 1899-01-28

**Authors:** 


					, CADBURY'S T-T ?M CADBURYS
GOOOA A M, OOCOA
ABSOLUTELY PURE. NO DRUGS OR ALKALIES USED
KT ^^WCiASQflir WEBTV* ImfSmaat
N?. 644. Vol. XXY. GfSSSJS] Saiubdat,
Jan. 28th, 1899.
\W1CU HOSP.LIAJ.
j-SubPoriptionTwrns.] pRICE TWOPENCE.

				

